# Wissenschaftliche Weiterbildung Älterer in Zeiten der COVID-19-Pandemie – Sichtweisen von Teilnehmenden an „Studieren ab 50“ der Universität Magdeburg

**DOI:** 10.1007/s40955-022-00221-x

**Published:** 2022-07-29

**Authors:** Annika Felix, Sarah Berndt, Jasmin Dabitz, Paul Schubert

**Affiliations:** Otto-von-Guericke-Universität Magdeburg, Magdeburg, Deutschland

**Keywords:** Wissenschaftliche Weiterbildung Älterer, Seniorenstudium, Weiterbildungsteilnahme, COVID-19-Pandemie, Academic continuing education for the elderly, Senior studies, Continuing education participation, COVID-19 pandemic

## Abstract

Der Beitrag untersucht die Wahrnehmung der COVID-19-Pandemie durch aktuelle und ehemalige Teilnehmende an wissenschaftlicher Weiterbildung Älterer am Beispiel des Programms „Studieren ab 50“ der Universität Magdeburg und fragt danach, inwieweit die COVID-19-Pandemie zu Benachteiligungen in der Bildungsteilhabe in diesem Bereich führt. Er nimmt damit zwei bislang nur wenig betrachtete Perspektiven auf die Auswirkungen der COVID-19-Pandemie im Bereich der Weiterbildung ein: a) die Sicht der Teilnehmenden und b) den Bereich der wissenschaftlichen Weiterbildung Älterer. Grundlage der Analysen bildet eine Befragung von derzeitigen und ehemaligen Teilnehmenden des Weiterbildungsangebots im Sommersemester 2021 (N 302, Rücklauf 33,3 %). Mittels (multinomial) logistischer Regressionsanalysen wird untersucht, womit die Weiterbildungsteilnahme in Zeiten der COVID-19-Pandemie in Zusammenhang steht. Es zeigt sich, dass hier vor allem teilnahmebezogene Faktoren, wie die Bewertung der bisherigen Teilnahmeerfahrungen und pandemiebezogene Aspekte, wie die Angst vor einer Ansteckung, eine Rolle spielen. Der Beitrag diskutiert die Ergebnisse vor dem Hintergrund der künftigen Ausgestaltung von wissenschaftlichen Weiterbildungsangeboten für Ältere.

## Abstract (en)

This article examines the perception of the COVID-19 pandemic by participants in post-professional academic continuing education using the example of the program “Studying over 50” at University of Magdeburg and asks to what extent the COVID-19 pandemic leads to disadvantages in educational participation in this area. It thus takes two perspectives on the effects of the COVID-19 pandemic in the field of continuing education that have been little considered so far: a) the view of participants and b) the field of post-professional scientific continuing education. The analyses are based on a survey of current and former participants in continuing education in the summer semester of 2021 (N 302, response rate 33.3%). (Multinomial) logistic regression analyses are used to investigate the factors that influence the participation in continuing education in times of the COVID-19 pandemic. It is shown that participation-related factors, such as the evaluation of previous participation experiences, and pandemic-related aspects, such as the fear of infection, play an important role. The article discusses the results in the context of the future design of post-professional scientific continuing education programs.

## Einleitung und Forschungsfrage

Für die Sicherung der gesellschaftlichen und sozialen Teilhabe nimmt die Bildung in allen Lebensphasen – so auch im Alter – eine zentrale Rolle ein. Es kann unterstellt werden, dass dieser Befund gerade in volatilen Zeiten bzw. Phasen der Transformation, wie aktuell unter den Bedingungen der COVID-19-Pandemie, von besonderer Bedeutung ist. Sowohl für die Bewältigung der COVID-19-Pandemie als auch für die Sicherung der Bildung über die ganze Lebensspanne ist folglich ein hoher Bildungsbedarf Älterer zu konstatieren (vgl. DGfE 2020, S. 3). Gleichzeitig ist der Bereich der Erwachsenen- und Weiterbildung selbst stark von pandemiebedingten Veränderungen betroffen und herausgefordert (vgl. DGfE 2020, S. 1). Die Auswirkungen der Pandemie auf diesen Bildungssektor lassen sich mittels der Begriffe „Disruption“, „Katalysator“ und „Brennglas“ umschreiben (Denninger und Käpplinger 2021, S. 174–175; Hoenig und Molzberger 2021, S. 156). Im Zuge des Lockdowns im Frühjahr 2020 und der dabei geltenden Kontaktbeschränkungen wurden die etablierten Angebote eingeschränkt oder eingestellt („Disruption“), programmatische Neu- und Weiterentwicklungen, insbesondere im Bereich der digitalen Formate, in deutlich stärkerem Maße als bisher erprobt („Katalysator“) oder bekannte Probleme, wie etwa eine unzureichende Finanzierung, nun besonders offenkundig („Brennglas“). Teils ist auch von „Kompensation“ die Rede (DGfE 2020, S. 1), wenn etwa digitale Angebote bisherige Präsenzformate ersetzen (mussten). Je nach Betrachtungsweise können die Veränderungen im Bereich der Erwachsenen- und Weiterbildung als „Wendepunkt, als Chance oder als Entscheidungsmoment“ (Hoenig und Molzberger 2021, S. 155) gedeutet werden.

Bisherige Untersuchungen zu den Auswirkungen und dem Umgang mit der COVID-19-Pandemie im Bereich der Erwachsenen- und Weiterbildung nehmen vor allem die Perspektive der Weiterbildungsanbieter ein, wie der Überblick von Denninger und Käpplinger (2021, S. 170) zeigt. Studien zu den Sichtweisen der Teilnehmenden entsprechender Angebote sind demgegenüber deutlich seltener und zumeist berufsbezogen (vgl. z. B. Kleinert et al. 2021a). Befunde zur wissenschaftlichen Weiterbildung Älterer^1^ im Kontext der COVID-19-Pandemie liegen bislang kaum vor. Erste Entwicklungen skizzieren etwa Gottl (2020), Lörcher und Weiss (2020), Dabo-Cruz et al. (2022), Göres (2022) sowie Lörcher et al. (2022).

Die Statistik der Gasthörenden deutscher Hochschulen verweist auf einen deutlichen Rückgang der Teilnehmenden in diesem Bereich. Waren vor Pandemiebeginn im Wintersemester (WiSe) 2019/20 noch knapp 15.500 Personen im Alter ab 65 Jahren als Gasthörende an deutschen Hochschulen eingeschrieben, so ist zum WiSe 2020/21 ein Rückgang auf nur noch rund 6500 Personen in dieser Altersgruppe zu verzeichnen (vgl. Statistisches Bundesamt 2021). Auch wenn in die Gasthörendenstatistik nicht alle Angebote der wissenschaftlichen Weiterbildung Älterer einfließen (vgl. Felix 2018, S. 8–9), so zeichnen sich doch deutliche Hinweise auf die disruptiven Auswirkungen der COVID-19-Pandemie ab (vgl. Göres 2022). Auch Dabo-Cruz et al. (2022, S. 39) führen an, dass die Zahl der Teilnehmenden an den Programmen der Universitäten Frankfurt am Main, Hamburg, Mainz und München im Zuge der COVID-19 Pandemie um etwa die Hälfte zurückgegangen ist.

Hier schließt der vorliegende Beitrag an. Anhand einer Befragung im Sommersemester (SoSe) 2021 von derzeitigen und ehemaligen Teilnahmenden des Programms „Studieren ab 50“ der Otto-von-Guericke-Universität Magdeburg wird untersucht, wie die Befragten die COVID-19-Pandemie wahrnehmen. Im Zentrum steht sodann die Frage, inwieweit soziodemografische, teilnahme- und kontextbezogene Aspekte mit der Weiterbildungs(nicht‑)teilnahme während der COVID-19-Pandemie und der künftigen Teilnahmeabsicht in Zusammenhang stehen. Dabei soll diskutiert werden, inwieweit die COVID-19-Pandemie im Bereich der wissenschaftlichen Weiterbildung Älterer zu (neuen) Benachteiligungen in der Bildungsteilhabe führt und welche künftigen Herausforderungen in diesem Kontext adressiert werden müssen.

## Theoretischer Rahmen und Forschungsstand

Das Spektrum möglicher Bildungsaktivitäten im Alter ist breit gefächert und reicht von formaler (zertifizierter) über non-formale (nicht zertifizierte, aber professionell organisierte) bis hin zu informeller Bildung (vgl. Kommission der europäischen Gemeinschaften 2000, S. 9) sowie von hochschulischen über außerhochschulische bzw. privatwirtschaftliche Anbieter bis hin zu selbst organisierten Bildungsinitiativen (vgl. Sommer et al. 2007, S. 11). Bildung im Alter ist mit zahlreichen Vorteilen verbunden: Ältere können sich besser auf gesellschaftliche und ökonomische Veränderungen einstellen, ihre Eigenständigkeit und Gesundheit besser erhalten und auch im hohen Alter ihre Persönlichkeit weiterentwickeln. Durch den demografischen Wandel, aber auch aufgrund der verbesserten Gesundheitssituation Älterer, dem insgesamt höheren Bildungsstand und einer zunehmenden Wissensintensität der Erwerbsarbeit (vgl. Autorengruppe Bildungsberichterstattung 2018), ist künftig von einer steigenden Bildungsbeteiligung im Alter auszugehen.

Zugleich gibt es zahlreiche Gründe, warum sich ältere Personen nicht bzw. kaum an Weiterbildungsaktivitäten beteiligen. Nach Wiest et al. (2017) resultiert die Weiterbildungsaktivität aus dem Zusammenspiel von individuellen Merkmalen und Gelegenheitsstrukturen. Werden die individuellen Merkmale näher betrachtet, die für die Weiterbildungs(nicht-)teilnahme von Bedeutung sind, so verweisen bisherige Untersuchungen vor allem auf die Bedeutung der individuellen Vorbildung (vgl. Schmidt-Hertha und Müller 2017; Wiest et al. 2017; Hoffmann et al. 2020; Bilger und Strauß 2021), wobei höher qualifizierte Personen eher an Weiterbildungen teilnehmen, als jene mit geringeren Qualifikationen. Darüber hinaus bedingen u. a. das Geschlecht, das Alter, der Erwerbsstatus, der Migrationshintergrund, die Wohnregion (Ost‑/Westdeutschland) und die Zufriedenheit mit dem Haushaltseinkommen die (Nicht‑)Teilnahme an Weiterbildungsaktivitäten (vgl. Bilger und Strauß 2021, S. 46).

Wird statt der Teilnahmeentscheidung die Fortsetzung der Teilnahme betrachtet, so kann die situative (Nicht‑)Passung zur Erklärung des Drop-outs herangezogen werden (vgl. Hoffmann et al. 2021). Drop-out beschreibt dabei ein Phänomen, „bei welchem Personen, die zu einer Weiterbildungsmaßnahme angemeldet sind und bis zu einem bestimmten Zeitpunkt an ihr teilnehmen, ihre Teilnahme vor regulärem Ende dieser Maßnahme einstellen“ (Hoffmann et al. 2019, S. 34). Auf Grundlage von NEPS-Daten erweisen sich das Geschlecht, die Muttersprache, das Haushaltseinkommen, die Kursdauer und der Bildungsabschluss der Mutter erklärungskräftig für den Weiterbildungsabbruch (vgl. Hoffmann et al. 2019, S. 39). Mittels problemzentrierter Interviews lassen sich sieben Typen der Nicht-Passung bei Drop-out in der Weiterbildung identifizieren, die sich aus dem Zusammenspiel der (intra- und inter-)individuellen und (intra- und inter-)institutionellen Ebene ergeben (vgl. Hoffmann et al. 2021, S. 251–253). Neben individuellen Merkmalen, die bei der Frage der (Nicht‑)Teilnahme von Weiterbildungen zumeist als erklärende Variablen herangezogenen werden, bezieht die Typologie auch Merkmale ein, die sich aus institutionellen Aspekten und ihrem Wechselspiel mit individuellen Merkmalen ergeben können. So berücksichtigt der Typ der „individuell-institutionellen Nicht-Passung“ etwa Erwartungen und Interessenlagen des Individuums und die Zufriedenheit mit der Weiterbildungsinstitution bzw. -aktivität (vgl. Hoffmann et al. 2021, S. 252).

Auf die nachberufliche Lebensphase bezogen zeigt Bödecker (2017) anhand einer Befragung von Interessierten und ehemaligen Teilnehmenden an den Jahreszeitenakademien und Arbeitskreisen „Forschendes Lernen“ an der Universität Ulm, dass immer mehr Seniorinnen und Senioren privat stark eingebunden und engagiert sind. Viele unterstützen ihre Familien, gehen Ehrenämtern oder Teilzeitbeschäftigungen nach. Als Teilnahmebarrieren wirken somit andere Interessen und Verpflichtungen. Entsprechend der Drop-out Typologie von Hoffmann et al. (2021, S. 251) könnte hier von einer lebenskontextuellen Nicht-Passung gesprochen werden, die auf veränderte Lebensumstände zurückgeht.

Inwiefern sich die COVID-19-Pandemie auf die Teilnahme bzw. Fortsetzung der Teilnahme an Weiterbildungen befördernd oder hemmend auswirkt, ist bislang nur wenig erforscht. Aufgrund der anhaltenden pandemischen Situation sind seit März 2020 Weiterbildungsangebote in Präsenz nur eingeschränkt bzw. unter Auflagen möglich (vgl. Infoweb Weiterbildung 2020). Ein Vergleich verschiedener Typen von Anbietenden legt die Vermutung nahe, dass wissenschaftliche Institutionen besser auf die veränderte Situation reagieren konnten als dies bei nichtwissenschaftlichen Einrichtungen der Fall war (vgl. Christ und Koscheck 2021, S. 4–5). Neben diesem Rückgang des Angebots sind weitreichende Stornierungen, d. h. ein deutlicher Rückgang der Nachfrage festzustellen (vgl. z. B. Weiterbildung Hessen e. V. 2020). Da auch nach dem Lockdown keine Rückkehr zum Normalbetrieb möglich war, konnten viele Angebote weiterhin nur mit geringeren Teilnehmendenzahlen oder nur als Online-Veranstaltungen umgesetzt werden, was sowohl eine wirtschaftliche als auch eine Herausforderung an die Anpassungsfähigkeit von Weiterbildungsanbietern darstellt (vgl. Christ und Koscheck 2021).

Die COVID-19-Pandemie und die damit einhergehende zunehmende Digitalisierung fordert auch die Teilnehmenden verstärkt hinsichtlich ihrer digitalen Kompetenzen heraus. Für die berufliche Nutzung von Online-Lernangeboten stellen etwa Kleinert et al. (2021b) einen Anstieg fest, wobei gleichzeitig verstärkte Bildungsungleichheiten zutage treten, indem durch die Ausweitung des Online-Lernens vor allem Hochgebildete profitieren. Neben den digitalen Kompetenzen könnte auch auf der Seite der Teilnehmenden die Wahrnehmung der COVID-19-Pandemie eine zentrale Rolle bei der Entscheidung für oder gegen eine (Fortsetzung der) Weiterbildungsteilnahme spielen. Erste Hinweise darauf liefern für die Gruppe der bis 64-Jährigen bspw. Bilger und Strauß (2021, S. 46) anhand von Daten des Adult Education Survey (AES). Sie identifizieren die selbst wahrgenommene Belastung durch die Corona-Pandemie als relevanten Prädiktor für die Weiterbildungsbeteiligung. Wird mindestens eine der betrachteten Situationen (Gesamtsituation, Arbeitssituation, finanzielle und familiäre Situation) als stark/äußerst belastend bewertet, so steigt die Wahrscheinlichkeit zur Weiterbildungsteilnahme.

## Methodisches Design

**Figure 1:**
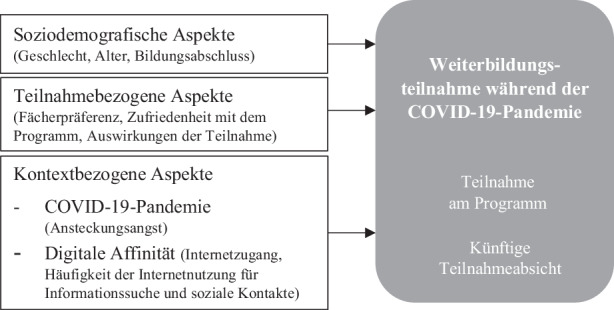

Der Beitrag untersucht die Wahrnehmung der COVID-19-Pandemie im Kontext der wissenschaftlichen Weiterbildung Älterer aus Sicht der Teilnehmenden von „Studieren ab 50“ der Universität Magdeburg und adressiert dabei folgende Fragen:

1. Wie nehmen die Befragten die COVID-19-Pandemie wahr?
2. Mit welchen soziodemografischen, teilnahme- und kontextbezogenen Aspekten steht die Weiterbildungsteilnahme während der COVID-19-Pandemie und die künftige Teilnahmeabsicht in Zusammenhang? (vgl. Abb. 1)

Bei „Studieren ab 50“ handelt es sich um ein Weiterbildungsangebot der Universität Magdeburg, welches im Zentrum für wissenschaftliche Weiterbildung (ZWW) angesiedelt ist. Das Programm richtet sich an ältere Erwachsene, soll ihnen u. a. den Zugang zu wissenschaftlichen Themen und Diskussionen eröffnen und im Rahmen gemeinsamer Studiermöglichkeiten jüngeren und älteren Studierenden die Gelegenheit zum Austausch bieten (vgl. OVGU 2022). Das Angebot lässt sich damit als „integriertes Seniorenstudium“ systematisieren, welches eine mittlere Strukturierung mit einer integrierten Vermittlung und einem vorhandenen Adressatenbezug kombiniert (vgl. Rathmann 2016a, S. 101). „Studieren ab 50“ ist an keine Zulassungsvoraussetzungen wie die Hochschulzugangsberechtigung oder an Altersgrenzen geknüpft und umfasst ausgewählte reguläre Lehrveranstaltungen der Fakultäten und Institute, spezielle Angebote für die Studienform „Studieren ab 50“ sowie Projektarbeitsgruppen. Ein Ablegen von Prüfungen ist nicht vorgesehen, auch gibt es keine vorab festgelegte Anzahl an zu belegenden Semesterwochenstunden (vgl. Freymark 2016; OVGU 2022). Die Anmeldung gilt jeweils für ein Semester. Seit der Gründung des Programms im Jahr 1991 verzeichnete sich ein stetiger Anstieg der Teilnehmenden, wobei durchgängig etwas mehr Frauen als Männer partizipierten und die Anmeldungen in den WiSe etwas höher ausfielen als in den SoSe (vgl. Rathmann 2016b, S. 12). Eine Programmanalyse zeigt, dass im Zeitraum 2008 bis 2015 auch die Anzahl der Veranstaltungsangebote insgesamt und besonders im Bereich der speziellen Angebote für die Studienform stetig zunahm (vgl. Weikert 2016, S. 75). Bisherige Befragungen im Rahmen von „Studieren ab 50“ verweisen auf einen hohen Anteil an „Stammteilnehmenden“, d. h. Personen, die sich wiederholt für mehrere aufeinander folgende Semester anmelden (vgl. Rathmann 2016c, S. 22). Zugleich zeigen sich eine hohe Zufriedenheit mit dem Programm, ein Interessenschwerpunkt der Teilnehmenden auf geschichtliche Themen sowie Teilnahmemotive ab, die sich dem Bereich der „Interessenverwirklichung/Bildung an sich“, d. h. einem genuinen Bildungsinteresse verorten lassen (vgl. Rathmann 2016c, S. 41, zur Systematisierung der Teilnahmemotive am Beispiel der Universität Hannover siehe auch: Schneider 2020, S. 75). Mit Beginn der COVID-19-Pandemie wurde das Angebot im SoSe 2020 ausgesetzt und ab dem WiSe 2020/21 mittels Online-Formaten fortgeführt. Im SoSe 2021 waren 128 Personen angemeldet. In den Semestern vor der Pandemie belief sich die durchschnittliche Teilnehmendenzahl auf rund 750 Personen pro Semester, im WiSe 2019/20 waren 701 Personen angemeldet (vgl. Paarmann und Roselli 2019, S. 20).

Die Datengrundlage der nachfolgenden Analysen bildet eine Befragung, in welche alle Personen einbezogen wurden, die im SoSe 2021 im Programm eingeschrieben waren oder innerhalb der vorangegangenen drei Jahre (SoSe 2018 bis WiSe 2020/21) daran teilnahmen. Die Untersuchung ist als Hybrid-Befragung konzipiert und kombiniert eine Online-Befragung mit einer postalischen Paper-Pencil-Untersuchung. Dieser Zugang wurde gewählt, da nicht alle Personen einen E‑Mail Account besitzen oder eine E‑Mail-Adresse bei der Anmeldung zum Programm angegeben hatten, über die sie kontaktiert werden konnten. Die Feldphase fand im Zeitraum Mai bis Juni 2021 statt. Nach Bereinigung der Daten liegen 302 gültige Fälle vor (Rücklauf 33,3 %). In der Gruppe der im SoSe 2021 Teilnehmenden fällt der Rücklauf mit 51,4 % deutlich höher aus, als bei den ehemaligen Teilnehmenden mit 29,8 %. Auch bezogen auf die beiden Modi der Datenerhebung ergeben sich Unterschiede. So konnte innerhalb der Paper-Pencil-Erhebung ein Rücklauf von 41,7 % erzielt werden, während dieser innerhalb der Online-Erhebung mit 31,8 % geringer ausfällt. 56,1 % der Befragten sind weiblich und 43,9 % männlich. Durchschnittlich sind die Teilnehmenden 72,5 Jahre alt. Vier Fünftel der Teilnehmenden (79,9 %), haben einen Hochschulabschluss erworben, ein Fünftel (20,1 %) verfügt nicht über einen akademischen Abschluss.

Die Wahrnehmung der COVID-19-Pandemie (Fragestellung 1) wird ausgehend von dem Instrument von Ohlbrecht et al. (2020) und in Anlehnung an Felix et al. (2022) erhoben und um zielgruppenspezifische Aussagen zu weiterbildungsbezogenen Aspekten erweitert (z. B. „Ich stecke mir weiterhin Ziele, auf die ich hinarbeite“). Zur Erklärung der Weiterbildungsteilnahme während der COVID-19-Pandemie und der künftigen Teilnahmeabsicht (Fragestellung 2) werden ausgehend von bisherigen Forschungsbefunden soziodemografische, teilnahmebezogene und kontextbezogene Aspekte einbezogen. Zu den betrachteten soziodemografischen Aspekten zählen das Geschlecht (weiblich/männlich), das Alter (in Jahren), sowie der Bildungsabschluss (ohne/mit Hochschulabschluss). Als teilnahmebezogene Aspekte werden die Fächerpräferenz (Interesse für Geschichte), die Zufriedenheit mit dem Programm und die Auswirkungen der Teilnahme^2^ herangezogen. Als kontextbezogene Aspekte gehen jene Variablen in die Analyse ein, die vor allem durch veränderte Rahmenbedingungen bzw. Anforderungen im Zuge der Pandemie von Relevanz sein könnten. Dazu zählt zum einen die Ansteckungsangst^3^ im Zuge der COVID-19-Pandemie und zum anderen die digitale Affinität, gemessen anhand des Internetzugangs (vorhanden/nicht vorhanden) und des Umfangs der Internetnutzung für Informationssuche und soziale Kontakte.^4^

## Ergebnisse

### COVID-19-Pandemie aus Sicht der derzeitigen und ehemaligen Teilnehmenden von „Studieren ab 50“

**Figure 2:**
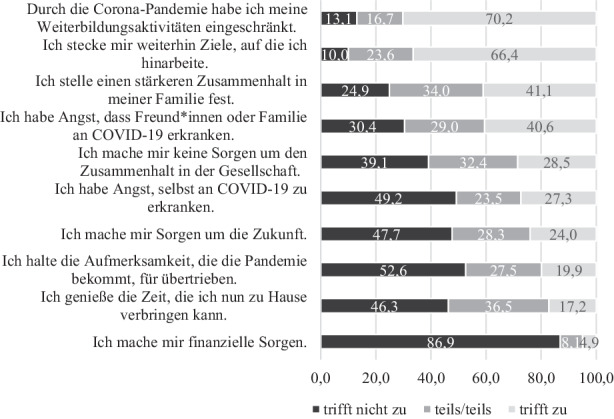

Die verschiedenen Aspekte der Auswirkungen der COVID-19-Pandemie sind für die Befragten in unterschiedlichem Maße relevant. So äußern die Befragten zuvorderst, dass sie aufgrund der Pandemie die eigenen Weiterbildungsaktivitäten eingeschränkt haben, gleichzeitig setzt sich jedoch die Mehrheit weiterhin Ziele, auf die sie hinarbeitet (vgl. Abb. 2).

Bezogen auf den Zusammenhalt in der eigenen Familie zeigt sich ein geteiltes Meinungsbild. So stellen rund vier von zehn Personen im Zuge der COVID-19-Pandemie einen stärkeren Zusammenhalt ihrer Familien fest, etwas mehr als ein Drittel bestätigt dies zum Teil und ein Viertel sieht keinen stärkeren Familienzusammenhalt. Die Angst vor einer eigenen Erkrankung an COVID-19 oder der Erkrankung naher Bezugspersonen fällt ebenfalls unterschiedlich aus. 40,6 % haben Angst, dass Freundinnen und Freunde oder Familienmitglieder an COVID-19 erkranken könnten; fast ein Drittel hat Angst, sich selbst zu infizieren.

Auch die Sorgen um den gesellschaftlichen Zusammenhalt sowie um die Zukunft zählen zu den Auswirkungen der COVID-19-Pandemie, die zumindest ein Teil der Befragten äußert. So geben zwei Fünftel an, beunruhigt wegen des allgemeinen Zusammenhalts der Gesellschaft zu sein. Auf 32,4 % trifft dies teilweise zu, während sich 28,5 % keine Sorgen machen. Ein Viertel sorgt sich zudem um die Zukunft. 28,3 % sind deshalb nur teilweise beunruhigt und fast die Hälfte hat keine Sorgen um die Zukunft. Etwa ein Fünftel hält die Aufmerksamkeit um die COVID-19-Pandemie für übertrieben, über die Hälfte hält sie für angemessen und 27,5 % bewegen sich dazwischen. Nur 17,2 % der Befragten genießen die Zeit, die sie zu Hause verbringen können, wiederum 46,3 % können diese Zeit nur bedingt oder gar nicht genießen. 36,5 % genießen die Zeit zu Hause teilweise. Finanzielle Aspekte spielen in der Zusammenschau nur eine untergeordnete Rolle. Lediglich 5 % der Befragten geben entsprechende Sorgen an.

### Weiterbildungsteilnahme während der COVID-19-Pandemie

**Figure 3:**
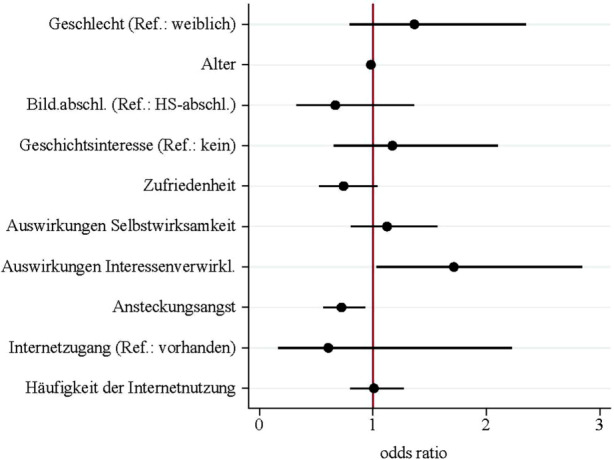

Rund die Hälfte der Befragten (51,4 %) hat während der COVID-19-Pandemie an „Studieren ab 50“ teilgenommen, während etwa die andere Hälfte (48,6 %) letztmalig im Zeitraum SoSe 2018 bis WiSe 2019/2020 partizipierte und seither (d. h. bis zum SoSe 2021) nicht mehr angemeldet ist. Es zeigt sich, dass die Weiterbildungsteilnahme in Zusammenhang mit den selbstberichteten Auswirkungen im Bereich „Interessenverwirklichung/Bildung an sich“ und der Ansteckungsangst steht (logistische Regression, Chi^2^(10) = 21,799; *p* = 0,016; *n* = 252). (Abb. 3).

**Figure 4:**
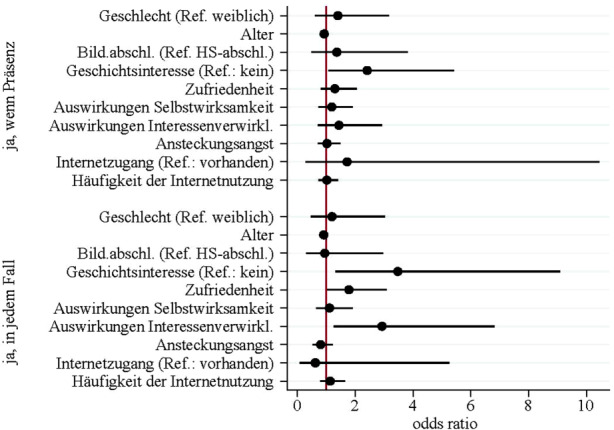

Je stärker im Verlauf der bisherigen Teilnahme positive Erfahrungen im Bereich der „Interessenverwirklichung/Bildung an sich“ als Aspekt des teilnahmebezogenen Bereichs gemacht wurden, umso höher ist die Chance, dass eine Teilnahme während der COVID-19-Pandemie erfolgt (odds ratio = 1,713). Die Ansteckungsangst als Aspekt des kontextbezogenen Bereichs steht hingegen mit der Weiterbildungsteilnahme in einem negativen Zusammenhang. Ein Anstieg der Ansteckungsangst um eine Einheit verringert dabei die Chance einer Teilnahme nach dem WiSe 2019/2020 um 28 % (odds ratio = 0,720; vgl. Abb. 4). Damit erweist sich lediglich jeweils ein Prädiktor aus dem teilnahmebezogenen sowie kontextbezogenen Bereich als relevant für die Teilnahme an den Semestern der COVID-19-Pandemie. Die betrachteten soziodemografischen Aspekte Geschlecht, Alter und Bildungsabschluss sind hingegen gänzlich ohne Bedeutung.

### Künftige Teilnahmeabsicht am Weiterbildungsprogramm

Die künftige Teilnahmeabsicht ist insgesamt relativ hoch ausgeprägt. So äußern 23,6 % der Befragten im nächsten Semester (WiSe 2021/22) auf jeden Fall teilnehmen zu wollen, weitere sechs von zehn Personen knüpfen ihre erneute Teilnahme an die Möglichkeit, dass wieder Präsenzveranstaltungen möglich sind. Lediglich 17,4 % der Befragten sind noch unschlüssig bzw. nicht an einer künftigen Teilnahme interessiert.

Die künftige Teilnahmeabsicht an „Studieren ab 50“ steht mit soziodemografischen und teilnahmebezogenen Aspekten in Zusammenhang (multinomial logistische Regression, Chi^2^(20) = 35,73; *p* = 0,017; *n* = 251). Für die Teilnahmeabsicht, die an die Voraussetzung eines Angebots von Präsenzveranstaltungen gekoppelt ist, erweisen sich das Alter und das Themeninteresse „Geschichte“ als relevant (vgl. Abb. 4). Mit steigendem Alter der Befragten sinkt dabei die Chance künftig eine Teilnahme unter der Bedingung von Präsenzveranstaltungen zu planen (im Vergleich zur geplanten Nichtteilnahme je Altersjahr um 8 %; odds rato = 0,920). Interessieren sich die Befragten für geschichtliche Themen, so steigt hingegen die Chance auf eine vorhandene Teilnahmeabsicht unter der Bedingung von Präsenzveranstaltungen (im Vergleich zu Personen ohne entsprechendes Interesse um 141 %; odds ratio = 2,414).

Für die unbedingte Teilnahmeabsicht (d. h. die geplante Teilnahme unabhängig vom Format) sind ebenfalls das Alter und das Interesse für Geschichte von Bedeutung. Mit steigendem Alter sinkt wiederum die Chance künftig teilnehmen zu wollen (im Vergleich zur geplanten Nichtteilnahme je Altersjahr um 9 %; odds ratio = 0,909). Ein Interesse für das Themengebiet Geschichte führt zu einer um 247 % höheren Chance künftig in jedem Fall eine Teilnahme an „Studieren ab 50“ zu planen (odds ratio = 3,473). Zusätzlich spielen zwei weitere Prädiktoren aus dem Bereich der teilnahmebezogenen Merkmale für die unbedingte Teilnahmeabsicht eine Rolle: die Zufriedenheit mit dem Programm und die Auswirkungen der Teilnahme im Bereich „Interessenverwirklichung/Bildung an sich“. Je höher dabei die Zufriedenheit mit der bisherigen Teilnahme ausfällt, umso höher ist auch die Chance einer beabsichtigten künftigen Teilnahme unabhängig vom Format der Veranstaltungen im Vergleich zur geplanten Nichtteilnahme. Je steigende Einheit der Zufriedenheit erhöht sich die Chance um 78 % (odds ratio = 1,781). Auch die wahrgenommenen Auswirkungen im Bereich „Interessenverwirklichung/Bildung an sich“ wirken sich positiv auf die künftige Teilnahmeabsicht aus. Je stärker positive Auswirkungen mit der bisherigen Teilnahme verknüpft werden, umso höher ist die Chance, künftig in jedem Fall an „Studieren ab 50“ teilnehmen zu wollen (193 %, odds ratio 2,926) anstatt sich gegen die Anmeldung zu entscheiden zu wollen.

## Diskussion

Der Beitrag rückt zwei bislang nur wenig betrachtete Sichtweisen auf die Auswirkungen der COVID-19-Pandemie im Bereich der Weiterbildung in dem Mittelpunkt, indem zum einen die Perspektive der Teilnehmenden und zum anderen der Bereich der wissenschaftlichen Weiterbildung Älterer am Beispiel von „Studieren ab 50“ der Universität Magdeburg betrachtet wird. Die deskriptiven Ergebnisse zeigen, dass die derzeitigen und ehemaligen Teilnehmenden mehrheitlich ihre Weiterbildungsaktivitäten im Zuge der Corona-Pandemie eingeschränkt haben, sich zugleich aber weiterhin Ziele setzen, auf die sie hinarbeiten. Bezogen auf Sorgen und Ängste, die mit der aktuellen Situation einhergehen können, wie etwa die Angst vor Ansteckung oder aber Sorgen um den gesellschaftlichen Zusammenhalt, zeigt sich ein geteiltes Meinungsbild. Mehrheitlich ablehnend stehen die Befragten hingegen der Einschätzung gegenüber, dass der Pandemie zu viel Aufmerksamkeit zuteilwerde.

Die Analysen zur Weiterbildungsteilnahme(-absicht) in Zeiten der COVID-19-Pandemie verdeutlichen, dass besonders Aspekte aus dem teilnahmebezogenen Bereich eine Rolle spielen. Je stärker die Befragten von positiven Auswirkungen hinsichtlich der „Interessenverwirklichung/Bildung an sich“ aufgrund der bisherigen Teilnahme berichten, desto eher nehmen sie während der COVID-19-Pandemie teil und beabsichtigen auch künftig – unabhängig vom Format der Veranstaltungen – eine Teilnahme. Zusätzlich stehen das Themeninteresse für Geschichte und teilweise die Zufriedenheit mit der künftigen Teilnahmeabsicht in Zusammenhang. Die Ergebnisse lassen sich vor dem Hintergrund der Relevanz der individuell-institutionellen Passung für die Entscheidung zur Fortsetzung von Weiterbildungsmaßnahmen (vgl. Hoffmann et al. 2021) deuten. Da die bisherigen Untersuchungen im Rahmen von „Studieren ab 50“ (vgl. Rathmann 2016c; Weikert 2016) zeigen, dass die Teilnehmenden vor allem aufgrund von Motiven im Bereich „Interessenverwirklichung/Bildung an sich“ teilnehmen und das Themenfeld der Geschichte den höchsten Zuspruch erhält, ist davon auszugehen, dass sich die Programmkonzeption verstärkt darauf ausrichtet und eine entsprechende individuell-institutionelle Passung die Teilnahme während der COVID-19-Pandemie und die künftige Teilnahmeabsicht begünstigt.

Zusätzlich zu den teilnahmebezogenen Aspekten erweist sich die Ansteckungsangst, als Aspekt des kontextbezogenen Bereichs, für die Teilnahme während der COVID-19-Pandemie von Bedeutung. Je stärker die Ansteckungsangst bei den Befragten ausgeprägt ist, umso geringer ist die Chance zur Teilnahme am Weiterbildungsangebot während der Pandemie. Hier könnten auch die Planungsunsicherheit im Zuge der Programmkonzeption und der Anmeldephase eine Rolle gespielt haben. Da aufgrund des dynamischen Infektionsgeschehens kaum verlässliche Planungen vor Semesterbeginn zum Format der Veranstaltungen möglich waren, verzichteten möglicherweise Personen mit hoher Ansteckungsangst auf eine Teilnahme. Die deskriptiven Befunde zeigen, dass sich die Ansteckungsangst der Befragten insgesamt im mittleren Bereich bewegt. Hier wäre in weiterführenden Untersuchungen näher zu betrachten, wovon diese abhängig ist und ob sich dadurch neue Ungleichheiten in der Bildungsteilhabe manifestieren. Gleichwohl deuten die Ergebnisse darauf hin, dass die COVID-19-Pandemie möglicherweise nur zu einem eher kurzfristigen Rückgang der Teilnehmenden führen könnte, da die Ansteckungsangst zwar für die aktuelle Teilnahme, nicht jedoch für die künftige Teilnahmeabsicht ein hemmender Faktor ist. Somit könnten die im Zuge der COVID-19-Pandemie ausgeschiedenen Personen künftig wieder für eine Teilnahme am Programm gewonnen werden.

Die Affinität gegenüber digitalen Medien steht in der vorliegenden Untersuchung nicht in Zusammenhang mit der Weiterbildungsteilnahme(-absicht). Hier ist zum einen die Erfassung der einbezogenen Konstrukte kritisch zu diskutieren. So wurde zur Messung der digitalen Affinität der Internetzugang und der Umfang der Internetnutzung im Bereich der Informationsbeschaffung und sozialen Kontakte (vgl. Kortmann et al. 2021) herangezogen, wodurch die Affinität gegenüber digitalen Medien nur sehr eingeschränkt erfasst werden konnte. Rückschlüsse auf die digitalen Kompetenzen oder die Einstellungen der Befragten gegenüber digitalen Medien sind demzufolge nicht möglich. Hier sollten künftige Untersuchungen eine breitere Erfassung anstreben. Zum anderen wurde nicht das tatsächliche Teilnahmeverhalten im jeweiligen Semester, für das sich die Befragten zuletzt angemeldet hatten, erfasst. So ist durchaus denkbar, dass eine Anmeldung unter der Prämisse erfolgte, dass Präsenzveranstaltungen möglich sein werden, wodurch Personen zu den „aktiven“ Teilnehmenden gezählt werden, die jedoch nicht an den Online-Angeboten während der Pandemie partizipierten. Auch hier könnte in weiterführenden Untersuchungen eine genauere Abfrage, etwa zur Anzahl und Art belegter Veranstaltungen, erfolgen.

Eine Relevanz soziodemografischer Aspekte lässt sich mit Ausnahme der Bedeutung des Alters für die künftige Teilnahmeabsicht anhand der vorliegenden Daten ebenfalls nicht nachzeichnen. Dies steht zunächst im Gegensatz zu bisherigen Forschungsbefunden (vgl. Schmidt-Hertha und Müller 2017; Wiest et al. 2017; Hoffmann et al. 2019, 2020; Bilger und Strauß 2021), welche individuelle Merkmale, vor allem die individuelle Vorbildung aber auch das Geschlecht, als relevant für die (Fortsetzung der) Weiterbildungsteilnahme herausstellen. So nehmen Frauen im Gegensatz zu Männern und Höhergebildete im Vergleich zu Personen ohne Hochschulabschluss zwar absolut betrachtet häufiger am Programm „Studieren ab 50“ teil, bezogen auf ihre Chance zur Teilnahme während der COVID-19-Pandemie und ihre künftige Teilnahmeabsicht unterscheiden sie sich jedoch nicht. Möglicherweise ist hierfür die in die vorliegende Untersuchung einbezogenen selektive Gruppe Älterer ausschlaggebend. Diese kennzeichnet sich durch eine hohe Bildungsaffinität, da sie in der Vergangenheit bereits in mindestens einem Semester am Bildungsprogramm „Studieren ab 50“ teilgenommen hat, was möglicherweise Unterschiede in der individuellen Vorbildung nivelliert. Interessant wäre hier der zusätzliche Einbezug einer Vergleichsgruppe von Personen, die bislang noch nicht am Angebot partizipiert haben. Das Alter der Befragten ist jedoch als relevant für die künftige Teilnahmeabsicht der Befragten einzuschätzen, wobei in Einklang mit bisherigen Untersuchungen die Teilnahmeabsicht mit steigendem Alter sinkt.

Die präsentierten Ergebnisse unterliegen verschiedenen Limitationen. So ist die realisierte Stichprobe eher gering und die Analysen damit mit einer hohen Unsicherheit behaftet. Aufgrund des Designs der Untersuchung kann zudem nicht ausgeschlossen werden, dass bestimmte Personen unter- oder überrepräsentiert sind. Auch verweist die Modellgüte der Regressionsanalysen darauf, dass ein Großteil relevanter Faktoren für die Weiterbildungsteilnahme(-absicht) nicht einbezogen wurde. Weiterhin wurde lediglich das Weiterbildungsangebot einer einzelnen Universität untersucht. Die Übertragbarkeit der Befunde ist daher, insbesondere aufgrund der hohen Variabilität der Programme (vgl. Rathmann 2016a) und dem standortspezifischen Umgang mit den pandemiebedingten Einschränkungen, nur begrenzt möglich. Bestehende Initiativen, die sich um eine standortübergreifende Datenlage in diesem Bereich bemühen, wie der Arbeitskreis Forschungsfragen und Statistik der Bundesarbeitsgemeinschaft wissenschaftliche Weiterbildung Älterer (vgl. Bertram et al. 2016; Lörcher und Lutz 2018), könnten künftig verstärkt die Aspekte der Digitalisierung in diesem Bereich und die Organisation der Angebote unter Pandemiebedingungen in den Blick nehmen und damit das bestehende Forschungsdesiderat adressieren.

Trotz aller Einschränkungen und Vorläufigkeit, denen die präsentierten Ergebnisse unterliegen, lässt sich zusammenfassend festhalten, dass im Kontext der COVID-19-Pandemie zum einen bisherige Aspekte, wie etwa die individuell-institutionelle Passung für die Weiterbildungsteilnahme(-absicht) bedeutsam bleiben, diese aber andererseits um neue Aspekte, wie die Ansteckungsangst, erweitert werden. Es bleibt abzuwarten, welche Rolle diese für die künftige Bildungsbeteiligung in diesem Bereich spielen werden, ob sich tatsächlich nur ein punktueller Zusammenhang abzeichnet oder ob längerfristige Auswirkungen auf die Teilhabechancen zu erwarten sind. Schließlich ist zu betonen, dass den Angeboten der wissenschaftlichen Weiterbildung Älterer, insbesondere auch unter den Bedingungen der COVID-19-Pandemie, eine wichtige Bedeutung für die soziale Teilhabe und den gesellschaftlichen Zusammenhalt zukommt und auch verstärkte Forschungsaktivitäten zur Sichtbarkeit entsprechender Angebote beitragen können.

## Footnotes

1 Der Bereich der wissenschaftliche Weiterbildung Älterer umfasst Angebote der Hochschulen, die sich an ältere Erwachsene richten (vgl. ausführlicher zu Entstehung: Bertram et al. 2017). Da nicht die berufliche Verwertbarkeit im Vordergrund steht und sich ein Großteil der Teilnehmenden in der nachberuflichen Lebensphase befindet, wird auch von nachberuflicher wissenschaftlicher Weiterbildung gesprochen. Die einzelnen Angebote sind sehr vielgestaltig und auch die Bezeichnungen variieren je nach Standort. Als Oberbegriff für Programme, die über eine allgemeine Gasthörerschaft hinausgehen und (zusätzlich oder ausschließlich) eigene Veranstaltungen anbieten, findet häufig der Begriff „Senior*innenstudium“ Verwendung (vgl. Felix 2018; Jütte und Lobe 2022, S. 7), wobei dieser durchaus kritisch diskutiert wird (vgl. Rathmann 2016a, S. 196-197).

2 Frageformulierung: „Welche persönlichen Erfahrungen haben Sie mit ‚Studieren ab 50‘ gemacht?“ 5‑stufiges Antwortformat von 1 „trifft überhaupt nicht zu“ bis 5 „trifft voll und ganz zu“. Eine Faktorenanalyse (Hauptkomponentenanalyse, erklärte Varianz 51,6 %) der acht Items (KMO-Kriterium = 0,73) erbringt eine 2‑Faktoren-Lösung. Bildung der Mittelwertindizes „Auswirkungen im Bereich Selbstwirksamkeit und Partizipation“ (4 Items, Cronbachs Alpha = 0,76) und „Auswirkungen im Bereich Interessenverwirklichung/Bildung an sich“ (4 Items, Cronbachs Alpha = 0,57). Die Bezeichnung der Indizes erfolgt in Anlehnung an Felix und Schneider (2022).

3 Frageformulierung: „Inwiefern treffen nachstehende mögliche Situationen oder Lebensumstände im Zuge der Corona-Pandemie auf Sie zu?“ 5‑stufiges Antwortformat von 1 „trifft überhaupt nicht zu“ bis 5 „trifft voll und ganz zu“. Bildung der Mittelwertindex der Items: „Ich habe Angst, selbst an COVID-19 zu erkranken“ und „Ich habe Angst, dass Freund*innen oder Familie an COVID-19 erkranken“ (Cronbachs Alpha = 0,74). Bivariate Analysen zeigen, dass von den zehn Items der Skala lediglich die Ansteckungsangst in Zusammenhang mit der Weiterbildungteilnahme steht, weshalb im Folgenden (Abschn. 4.2 und 4.3) ausschließlich dieser Aspekt der Wahrnehmung der COVID-19-Pandemie einbezogen wird.

4 Frageformulierung: „Wie häufig nutzen Sie das Internet für folgende Zwecke?“, 5‑stufiges Antwortformat von 1 „täglich“ bis 6 „nie“ (vgl. Vogel et al. 2021, S. 11, siehe auch Kortmann et al. 2021). Bildung Mittelwertindex der Items „Kontakt mit Freund*innen, Bekannten und Verwandten (z. B. E‑Mail, Facebook, Chat, Videotelefonie z. B. Skype)“ und „Suche nach Informationen (z. B. Nachrichten, Ratgeberseiten)“ (Cronbachs Alpha = 0,84), da davon ausgegangen wird, dass vor allem diese beiden Aspekte relevant für die Teilnahme an Online-Veranstaltungen sein könnten.

## References

[CR1] Autorengruppe Bildungsberichterstattung (2018). Bildung in Deutschland 2018: Ein indikatorengestützter Bericht mit einer Analyse zu Wirkungen und Erträgen von Bildung.

[CR3] Bertram, T., Lechner, D., Rathmann, A., & Weigert, Y. (2016). Aktuelle Forschungsaktivitäten zu einer systematischen statistischen Erfassung der universitären Bildung für Ältere – Einblicke und Ausblicke. Vorseminar. In *Jahrestagung der Deutschen Gesellschaft für Weiterbildung und Fernstudien (DGWF)*. Universität Wien, 14. Sept. 2016. https://dgwf.net/files/web/ueber_uns/jahrestagungen/2016/DGWF-Jahrestagung2016_Abstractband.pdf. Zugegriffen: 19. Juli 2022.

[CR2] Bertram T, Dabo-Cruz S, Pauls K, Vesper M, Hörr B, Jütte W (2017). Bundesarbeitsgemeinschaft Wissenschaftliche Weiterbildung für Ältere (BAG WiWA). Weiterbildung an Hochschulen. Der Beitrag der DGWF zur Förderung wissenschaftlicher Weiterbildung.

[CR4] Bilger, F., & Strauß, A. (2021). *Weiterbildungsverhalten in Deutschland 2020. Ergebnisse des Adult Education Survey – AES Trendbericht*. Berlin: Bundesministerium für Bildung und Forschung. https://www.bmbf.de/SharedDocs/Publikationen/de/bmbf/1/31690_AES-Trendbericht_2020.pdf?__blob=publicationFile&v=4. Zugegriffen: 19. Juli 2022.

[CR5] Bödecker F (2017). Was Senioren davon abhält, an wissenschaftlicher Weiterbildung teilzunehmen. „Ich habe viele andere Verpflichtungen und Interessen“. DIE Zeitschrift für Erwachsenenbildung.

[CR6] Christ, J., & Koscheck, S. (2021). Auswirkungen der Corona-Pandemie auf Weiterbildungsanbieter. Vorläufige Ergebnisse der wbmonitor Umfrage 2020. Bundesinsitut für Berufsbildung. https://www.vsbi.de/media/Aktuelles/2021/21-01-21_Umfrage_wb-monitor/21-01-21_Umfrage_Auswirkungen_Corona-Pandemie.pdf. Zugegriffen: 19. Juli 2022.

[CR7] Dabo-Cruz S, Lörcher B, Lutz K, Pauls K (2022). Senior*innenstudium digital – Herausforderungen und neue Perspektiven. Zeitschrift Hochschule und Weiterbildung.

[CR8] Denninger A, Käpplinger B (2021). COVID-19 und Weiterbildung – Überblick zu Forschungsbefunden und Desideraten. Zeitschrift für Weiterbildungsforschung.

[CR9] DGfE – Deutsche Gesellschaft für Erziehungswissenschaft, Sektion Erwachsenenbildung (2020). Der Sektionsvorstand Erwachsenenbildung betont die Rolle der Erwachsenen- und Weiterbildung in der Bewältigung der Corona-Krise. https://www.dgfe.de/fileadmin/OrdnerRedakteure/Sektionen/Sek09_ErwB/2020_Corona-Zwischenruf_Sektion_Erwachsenenbildung.pdf. Zugegriffen: 19. Juli 2022.

[CR10] Felix, A. (2018). Akademische Bildung im Alter – ein Überblick zum Forschungsfeld. Enzyklopädie Erziehungswissenschaft Online. https://www.beltz.de/fachmedien/erziehungswissenschaft/enzyklopaedie_erziehungswissenschaft_online_eeo/artikel/38685-akademische-bildung-im-alter-ein-ueberblick-zum-forschungsfeld.html. Zugegriffen: 19. Juli 2022.

[CR12] Felix A, Schneider B (2022). Motive, Auswirkungen und Bilanzierung im nachberuflichen Studium. Zeitschrift für Hochschule und Weiterbildung.

[CR11] Felix A, Berndt S, Anacker J, Angenent H, Petri J, Zimenkova T (2022). Corona-Pandemie-Resilienz und ihre Bedeutung für Studienalltag und Studienerfolg – empirische Analysen und organisationale Ableitungen anhand des Studierendenpanels der Universität Magdeburg. Hochschulen in der Pandemie. Impulse für eine nachhaltige Entwicklung von Studium und Lehre.

[CR13] Freymark O, Freymark O (2016). Die Entstehung und Ausgestaltung des Kontaktstudiums „Studieren ab 50“ an der Otto-von-Guericke-Universität Magdeburg. Wissenschaftliche Weiterbildung für Ältere. Vergangenheit – Gegenwart – Zukunft, Festschrift zum 25-Jährigen Bestehen von Studieren ab 50.

[CR14] Göres, J. (2022). Digitales Senioren-Studium. Senioren studieren online weiter, Sueddeutsche Zeitung, online vom 13.03.2022. https://www.sueddeutsche.de/karriere/senioren-studium-e-learning-online-hochschule-1.5542759. Zugegriffen: 19. Juli 2022.

[CR15] Gottl, R. (2020). Per Videokonferenz ins Lieblingsseminar, Sueddeutsche Zeitung, online vom 06.11.2020. https://www.sueddeutsche.de/karriere/seniorenstudium-per-videokonferenz-ins-lieblingsseminar-1.5102499. Zugegriffen: 19. Juli 2022.

[CR16] Hoenig K, Molzberger G (2021). Erwachsenen- und Weiterbildung unter Pandemiebedingungen. Herausforderungen und Perspektiven. Zeitschrift für Weiterbildungsforschung.

[CR18] Hoffmann S, Thalhammer V, von Hippel A, Schmidt-Hertha B (2019). Drop-Out in der Weiterbildung – eine Verschränkung von Perspektiven zur (Re‑)Konstruktion des Phänomens Drop-Out. Zeitschrift für Weiterbildungsforschung.

[CR17] Hoffmann M, Wiest M, Widany S, Kaufmann-Kuchta K, Schrader J, Ioannidou A, Blossfeld H-P (2020). Der Beitrag non-formaler Bildungsbeteiligung für Lebenszufriedenheit älterer Erwerbstätiger. Monetäre und nicht monetäre Erträge von Weiterbildung.

[CR19] Hoffmann S, Thalhammer V, von Hippel A, Schmidt-Hertha B (2021). Situative (Nicht‑) Passung als Erklärungsansatz von Drop-Out in der Weiterbildung. Zeitschrift für Weiterbildungsforschung.

[CR20] Infoweb Weiterbildung (2020). Informationen zu Weiterbildungsangeboten in Deutschland. Status der Einschränkungen von Bildungsveranstaltungen aufgrund der Coronapandemie. https://edubase.org/service/BV_nach_BL.pdf. Zugegriffen: 19. Juli 2022.

[CR21] Jütte W, Lobe C (2022). Stichwort: Hochschulweiterbildung und Alter(n). Zeitschrift Hochschule und Weiterbildung.

[CR22] Kleinert, C., Vicari, B., Zoch, G., & Ehlert, M. (2021a). Wer bildet sich in Pandemiezeiten beruflich weiter? Veränderungen in der Nutzung digitaler Lernangebote während der Corona-Krise. Bericht. NEPS Corona & Bildung. https://www.lifbi.de/Portals/13/Corona/NEPS_Corona-und-Bildung_Bericht_7-Weiterbildung.pdf. Zugegriffen: 19. Juli 2022.

[CR23] Kleinert C, Zoch G, Vicari B, Ehlert M (2021). Work-related online learning during the COVID-19 pandemic in Germany. Zeitschrift für Weiterbildungsforschung.

[CR24] Kommission der europäischen Gemeinschaften (2000). Memorandum über Lebenslanges Lernen, Arbeitsdokument der Kommissionsdienststellen vom 30.10.2000. www.hrk.de/uploads/tx_szconvention/memode.pdf. Zugegriffen: 19. Juli 2022.

[CR25] Kortmann L, Hagen C, Endter C, Riesch J, Tesch-Römer C (2021). Internetnutzung von Menschen in der zweiten Lebenshälfte während der Corona-Pandemie: Soziale Ungleichheiten bleiben bestehen. dza Aktuell.

[CR26] Lörcher B, Lutz K (2018). Bildung Älterer an Hochschulen – Der Musterfragebogen des BAG WiWA Arbeitskreis Forschungsfragen &amp; Statistik. Vortrag. Jahrestagung der Bundesarbeitsgemeinschaft Wissenschaftliche Weiterbildung für Ältere (BAG WiWA).

[CR27] Lörcher B, Weiss E, Münchner Universitätsgesellschaft (2020). Zwei Semester digitale Lehre am Zentrum Seniorenstudium, zwei Semester mit Moodle, Zoom und Co. Jahresbericht 2020 der Münchener Universitätsgesellschaft.

[CR28] Lörcher B, Tippelt R, Weiss E (2022). Wissenschaftsbasiertes Lernen und digitaler Wandel im Seniorenstudium. Zeitschrift Hochschule und Weiterbildung.

[CR29] Ohlbrecht H, Anacker J, Jellen J, Lange B, Weihrauch S (2020). Zu den Auswirkungen der Corona-Pandemie auf das subjektive Wohlbefinden und die Alltagsbewältigung – Ergebnisse einer Online-Befragung.

[CR30] OVGU – Otto-von-Guericke-Universität Magdeburg (Hrsg.). (2022). Wissenschaftliche Weiterbildung an der Otto-von-Guericke-Universität Magdeburg, Broschüre Studieren ab 50, Sommersemester 2021, Magdeburg: Otto-von-Guericke-Universität Magdeburg. https://www.ovgu.de/unimagdeburg_media/Weiterbildung+_+Career+Service/Weiterbildung/Dokumente/Studieren+ab+50/Programm_Studieren_ab_50_SS2022-download-1-p-98014.pdf. Zugegriffen: 19. Juli 2022.

[CR31] Paarmann Y, Roselli A (2019). Weiterbildung al Third Mission: Das Programm „Studieren ab 50“ der OVGU Magdeburg.

[CR32] Rathmann A (2016). Alter(n)sbilder und Bildung im Alter an Hochschulen. Empirische Untersuchung von organisationalen und individuellen Alter(n)sbildern im Kontext der nachberuflichen wissenschaftlichen Weiterbildung in Deutschland.

[CR33] Rathmann A, Freymark O (2016). Struktur und Entwicklung der Teilnehmenden von „Studieren ab 50“. Wissenschaftliche Weiterbildung für Ältere. Vergangenheit – Gegenwart – Zukunft, Festschrift zum 25-Jährigen Bestehen von Studieren ab 50.

[CR34] Rathmann A, Freymark O (2016). Sichtweisen von Seniorenstudierenden und Regelstudierenden zum „Studieren ab 50“ im Sommersemester 2014 – Ergebnisse zweier Befragungen. Wissenschaftliche Weiterbildung für Ältere. Vergangenheit – Gegenwart – Zukunft, Festschrift zum 25-Jährigen Bestehen von Studieren ab 50.

[CR35] Schmidt-Hertha B, Müller M (2017). Bedeutung von bildungsbiografischen Zäsuren für die Weiterbildungsbeteiligung älterer Erwerbstätiger. Berufsbildung in Wissenschaft und Praxis.

[CR36] Schneider B (2020). Situation und Bildungsbedürfnisse von Teilnehmenden der nachberuflichen wissenschaftlichen Weiterbildung an deutschen Hochschulen am Beispiel des Gasthörenden- und Seniorenstudiums an der Leibniz Universität Hannover.

[CR37] Sommer C, Künemund H, Kohli M (2007). Zwischen Selbstorganisation und Seniorenakademie. Die Vielfalt der Altersbildung in Deutschland.

[CR38] Statistisches Bundesamt (2021). Statistik der Gasthörer. Gasthörer nach Semester, Nationalität, Geschlecht und Altersgruppen. Tabelle. https://www-genesis.destatis.de/genesis/online/link/tabelleErgebnis/21331-0001. Zugegriffen: 19. Juli 2022.

[CR39] Vogel C, Wettstein M, Klaus D, Spuling S, Kortmann L, Alcántara AL, Engstler H, Huxhold O, Nowossadeck S, Romeu Gordo L, Simonson J, Tesch-Römer C (2021). Deutscher Alterssurvey (DEAS): Fragebogen der DEAS-Kurzbefragung 2020.

[CR40] Weikert F, Freymark O (2016). Qualitätsverbesserungen – Erkenntnisse und Maßnahmen. Wissenschaftliche Weiterbildung für Ältere. Vergangenheit – Gegenwart – Zukunft, Festschrift zum 25-Jährigen Bestehen von Studieren ab 50.

[CR41] Weiterbildung Hessen e. V. (2020). Auswirkungen der COVID-19-Pandemie auf die hessischen Weiterbildungseinrichtungen. Ergebnisse einer Blitzumfrage von Weiterbildung Hessen e. V. https://weiterbildunghessen.de/fileadmin/Bilder/Presse/200430_Befragung_Weiterbildung_Hessen.pdf. Zugegriffen: 19. Juli 2022.

[CR42] Wiest M, Hoffmann M, Widnay S, Kaufmann K (2017). Trends in non-formaler Bildungsbeteiligung in der zweiten Lebenshälfte. Steigende Bildungsbeteiligung im Ruhestand. Zeitschrift für Gerontologie und Geriatrie.

